# The influence of cognitive ability in Chinese reading comprehension: can working memory updating change Chinese primary school students’ reading comprehension performance?

**DOI:** 10.3389/fpsyg.2023.1283781

**Published:** 2023-09-19

**Authors:** Jiacheng Gao, Zimo Yang, Fengjuan Li, Bahtinsagul Yasen, Suxia Wen

**Affiliations:** ^1^Xinjiang Key Laboratory of Mental Development and Learning Science, College of Psychology, Xinjiang Normal University, Urumqi, China; ^2^Department of Psychology, Fudan University, Shanghai, China; ^3^Department of Psychology, Southwest University, Chongqing, China

**Keywords:** working memory, central executive, Chinese reading comprehension, updating, primary school

## Abstract

With the development of educational cognitive neuroscience, language instruction is no longer perceived as mechanical teaching and learning. Individual cognitive proficiency has been found to play a crucial role in language acquisition, particularly in the realm of reading comprehension. The primary objective of this study was to investigate two key aspects: firstly, to assess the predictive effects of the central executive (CE) on the Chinese reading comprehension scores of Chinese primary school students, and secondly, to explore the influence of CE training on the Chinese reading comprehension performance of Chinese primary school students. Chinese primary school students were recruited as participants. Experiment 1 used a Chinese N-back task, a Chinese Stroop task, and a number-pinyin conversion task to investigate the predictive effect of the CE components on Chinese reading comprehension. Experiment 2, based on the results of Experiment 1, used the Chinese character N-back training to explore the influence of updating training on Chinese reading comprehension. The findings from Experiment 1 underscored that CE had a predictive effect on Chinese reading comprehension scores. And updating had a prominent role in it. Experiment 2 revealed that the experimental group exhibited an enhancement in their updating performance following N-back training. Although the reading comprehension performance of the two groups after training did not produce significant differences in total scores, the experimental group showed maintained and higher microscopic reading comprehension scores than the control group in the more difficult post-test. In summary, this study yields two primary conclusions: (1) CE was able to predict Chinese reading comprehension scores. Updating has an important role in prediction. (2) Updating training enhances students’ updating performance and positively influences students’ Chinese microscopic reading comprehension performance.

## 1. Introduction

Working memory (WM) is an essential human cognitive ability. The central executive (CE) is the core component of WM, a control system with limited attentional resources, and coordinates other components of WM (Baddeley and Hitch, 1974). Researchers have separated CE and found that there are three independent and moderately related components of CE: updating, inhibition, and shifting (Miyake et al., 2000). CE plays an important role in individual reading comprehension (Skehan, 1998, 2002; Giofrè et al., 2018; Shin, 2020; Zaccoletti et al., 2023).

Lower levels of CE are more likely to result in reading comprehension problems (Abu-Rabia, 2003; Nicolielo-Carrilho et al., 2018; Brunfaut et al., 2021; Linares and Pelegrina, 2023). However, most previous studies have focused on the role of CE in reading comprehension of phonetic characters, as represented by English. Little attention has been paid to the role of CE in reading comprehension of ideographic characters, represented by Chinese. However, there are significant differences between reading comprehension of ideographic and phonetic characters in both the acquisition and use processes. Further exploration of the role of WM in the reading comprehension of ideographic characters would be beneficial in refining the model of the role of WM in the process of reading comprehension. In addition, the CE developmental critical period occurs mainly during the individual school-age period (Welsh et al., 1991). The results of previous studies based on the critical period of CE have also confirmed the positive transfer effect of CE (Loosli et al., 2012; Ang et al., 2015; Artuso et al., 2019). Students in grades 4–5 are in the middle-high learning transition period. During this period, reading comprehension requires further acquisition of the ability to analyze articles, grasp their main ideas, and infer their meaning. At this time, reading comprehension activities rely heavily on WM.

Currently, with the development of educational neuroscience, research has found that general cognitive factors represented by WM are closely related to individual language learning (Wen, 2012; Van der Steen et al., 2017; Lukasik et al., 2018; Siu et al., 2018; Mavrou, 2020; Shin, 2020; Deldar et al., 2021; Vasylets and Marín, 2021; Fresneda, 2022; Kim, 2023).

Some studies have explored the role of WM on reading comprehension in children with learning disabilities and found a positive correlation between WM level and reading comprehension (Nicolielo-Carrilho et al., 2018). Studies based on normal participants have also confirmed the correlation between WM and reading comprehension (Peng et al., 2018; Shin et al., 2019; Hijikata and Koizumi, 2021; Huang et al., 2022; Orsolini et al., 2023). Loosli et al. (2012) conducted WM training with normal children and found that the training improved their English reading comprehension. Artuso et al. (2019) conducted WM updating training with Italian primary school students and found that the participants’ reading comprehension scores were significantly improved after the training. Several studies have examined the effects of WM training on the reading comprehension abilities of Chinese children with learning disabilities and similarly found positive effects of the training (Luo et al., 2013; Yang et al., 2017; Siu et al., 2018).

The purpose of the present study was to examine the predictive effects of CE of WM on Chinese reading comprehension scores of Chinese primary school students and how CE component training can influence reading comprehension performances. To solve this problem, we designed two experiments. Experiment 1 used Gao (2019) revised Chinese N-back task, Chinese Stroop task, and number-pinyin conversion task to examine the predictive effects of CE updating, inhibition, and shifting on primary school students’ Chinese reading comprehension scores. Experiment 2 was based on the results of Experiment 1 using Gao et al. (2023) revised Chinese character N-back training task for primary school students to investigate the influence of training on Chinese reading comprehension performance of Chinese primary school students.

Based on the limited extant research, we proposed two hypotheses. First, updating, inhibition, and shifting all predict Chinese reading comprehension scores of Chinese primary school students. Among them, updating reflects the continuous stability during the continuous processing of WM, and therefore plays a prominent role. Second, training on updating will have a positive influence on the Chinese reading comprehension performance of Chinese primary school students.

## 2. Experiment 1: the predictive effect of CE on Chinese reading comprehension scores of Chinese primary school students

### 2.1. Materials and methods

#### 2.1.1. Participants

We recruited 20 participants in the top 25% (high reading comprehension level group) and 20 participants in the bottom 25% (low reading comprehension level group) of grades 4–5 within a Chinese primary school based on their score ranking in the reading comprehension section of the previous semester’s Chinese final exam. A total of 40 participants (24 boys and 16 girls) were recruited. The participants’ age was 10.73 years (*SD* = 0.64). The total score for the reading comprehension section was 100. The difference in scores between the high level group (*M* = 70.10, *SD* = 10.07) and the low level group (*M* = 34.21, *SD* = 13.53) was significant [*t*_(38)_ = 9.57, *p* < 0.001, Cohen’s *d* = 3.01, *r* = 0.83]. All participants were from the same neighborhood school, had similar language use experiences and similar living environments, were right-handed, and had no similar experimental experiences. None of the students participating in the experiment had psychiatric, neurological, or developmental disorders, according to previous assessments by the medical and mental health departments of the school. The participants voluntarily took part in the experiment and signed written informed consent forms. Written consent was obtained from the students’ parents and the school before the experiment. The experiment was conducted for the period from November 15 to 20, 2021.

#### 2.1.2. Experimental design

A between-participants design was used for the experiment. Both high and low reading comprehension score groups were required to complete an updating task, an inhibition task, and a shifting task and record the accuracy. The experiment was completed in a double-blind experimental setting with a 1-day interval between tasks.

#### 2.1.3. Stimulus materials

To match the Chinese reading comprehension process, the updating task used the Chinese N-back task, which is more appropriate for the Chinese reading comprehension characteristics (Gao, 2019). The inhibition task used a Chinese Stroop character-color task adapted from the classic Stroop task (Spinks et al., 2000). The shifting task was adapted from Rogers and Monsell (1995) number-alphabet conversion task to a number-pinyin conversion task. Because some participants (fourth grade) had not fully acquired vowel and consonant discrimination, the alphabetic material was partially replaced with Chinese pinyin material.

#### 2.1.4. Procedures

(1) Chinese character updating N-back task

Harvey et al. (2004) found that the 1-back task is a pure updating task, so the whole Chinese character N-back task contains only two levels, 0-back and 1-back. The 0-back task requires judgments on Chinese characters “书” or “车.” The 1-back task requires judging whether the current presented Chinese character is consistent with the last presented Chinese character and responded with a key press. The 0-back and 1-back tasks each contain one block of five practice trials with feedback. At the end of the practice session, the participant can choose to practice again or go to the formal task. There was no feedback on the formal task. The 0-back formal task consisted of one block of 20 trials for the character “书” and 12 trials for the character “车.” The 1-back formal task consisted of three blocks of 96 trials. Each block had 20 trials of consistent judgments and 12 trials of inconsistent judgments. The accuracy of the participants was recorded.

(2) Chinese inhibition Stroop task

According to Wen and Li (2007), a Chinese Stroop word-color task was used. Chinese characters of different colors were presented in the experiment, and participants were asked to judge whether the color of the character’s meaning was consistent with the color of the character presented and responded with a keypress. There were three types of judgments: First, the color of the character matches the presented color. For example, the character “红(red)” is presented in red color. Second, the color of the meaning and the color of the presented character do not match. For example, “绿(green)” is presented in red. Third, the meaning of the character has no color meaning. For example, “师(teacher)” is presented in green color. The task starts with a block of five practice trials containing feedback. At the end of the practice session, the participant could choose to practice again or go to the formal task. There is no feedback on the formal task. Each block had 16 trials in which the color of the character was consistent with the presented color, 13 trials in which it was inconsistent, and three trials in which there was no color meaning. There were three blocks of 96 trials.

(3) Shifting number-pinyin conversion task

In the number-pinyin conversion task, a number, a pinyin, or a combination of a number and a pinyin is presented in blue or red. For example, “8b” in blue. Participants were required to make judgments based on the color and content of the material and responded with a key press. There are three types of judgments: First, when a blue number-pinyin combination is presented, the participant has to judge whether the number in the combination is odd or even. Second, when a red number-pinyin combination is presented, the participant needs to judge whether the pinyin in the number-pinyin combination is any of the single rhymes “a, o, e, i, u, ü.” Third, when a number or a pinyin is presented in red or blue alone, the participant has to ignore the presentation color and judge the number or pinyin directly. The task started with a block of five practice trials with feedback. At the end of the practice session, the participant could choose to practice again or go to the formal task. There was no feedback on the formal task. Each conversion block contained eight trials of the blue combination and eight trials of the red combination. The non-conversion task contained eight trials of independent numerical judgments and eight trials of independent pinyin judgments. Total three blocks, 96 trials.

### 2.2. Results

The accuracy of the participants’ three tasks was selected as the indicator, and the measurement scores of all participants were within 3 *SDs*, no data could be deleted. The accuracy rates of the participants with high and low Chinese reading comprehension scores in each task were shown in Table 1. The score of the high-level Chinese reading comprehension group was higher than the low-level group on all three tasks (*ps* < 0.05).

**TABLE 1:** Central executive (CE) tasks accuracy rates of two groups in Chinese reading comprehension (*M* ± *SD*).

Group	High scores group	Low scores group	*t*	Cohen’s *d*	*r*
Updating	0.71 ± 0.19	0.53 ± 0.14	3.42**	1.08	0.48
Inhibition	0.79 ± 0.17	0.68 ± 0.17	2.18*	0.65	0.31
Shifting	0.46 ± 0.19	0.32 ± 0.12	2.75**	0.88	0.40

*N* = 40.

*p < 0.05; **p < 0.01.

Participant type was targeted, and updating, inhibition, and shifting task accuracy were the independent variables for constructing the model. A logistic regression analysis was conducted by the forward selection method (Wald). The results of the analysis revealed the predictive effect of the CE components on primary school students’ Chinese reading comprehension scores (Table 2). The logistic regression model was significant (χ^2^ = 10.54, *p* = 0.001, -2 *log likelihood* = 44.81). The *Hosmer and Lemeshow* test was good (χ^2^ = 4.84, *p* = 0.77). The correct prediction rate of the model for the high Chinese reading comprehension score group was 61.9%. The correct prediction rate of the model for the low-score group was 73.7%. The correct prediction rate of the model for all participants was 67.5%. Among the variables included in the model, updating had a significant effect on Chinese reading comprehension scores (β = 6.64, *OR* = 763.31, *p* = 0.007). in the inhibition task and shifting task did not play a role in the model (*ps* > 0.05).

**TABLE 2:** Results of the first iteration of logistic regression of CE on scores in Chinese reading comprehension.

Factor	β	*SE*	Wald	*p*	*OR*
Updating accuracy	6.64	2.45	7.37	0.007	763.31**

**p < 0.01.

The experimental results suggest that CE has an important role in Chinese reading comprehension learning for Chinese primary school students. The findings are consistent with the results of the current study exploring the correlation between reading comprehension and WM (Artuso et al., 2019). The present study further refined the role of the CE components on Chinese reading comprehension and verified that updating plays an important role in Chinese reading comprehension (Hypothesis 1).

## 3. Experiment 2: the influence of updating training on Chinese reading comprehension score of Chinese primary school students

### 3.1. Materials and methods

#### 3.1.1. Participants

Seventy-three students in two natural classes of fourth grade within a Chinese primary school were re-recruited. The age was 10.06 years (*SD* = 0.60). The two classes were randomly divided into experimental and control groups, with 35 students in the experimental group (18 boys and 17 girls) and 38 students in the control group (18 boys and 20 girls). All participants were from the same community school, had similar language use experience and living environment, were right-handed, and had no similar experimental experience. None of the students participating in the experiment had psychiatric, neurological, or developmental disorders, according to previous assessments by the medical and mental health departments of the school. The participants voluntarily took part in the experiment and signed written informed consent forms. Written consent was obtained from the students’ parents and the school before the experiment. The experiment was conducted for the period November 22, 2021 to January 5, 2022.

#### 3.1.2. Experimental design

A between-participants design was used for the experiment. The independent variable was whether or not to receive updating training. The dependent variable was the Chinese reading comprehension scale score. All participants received normal school instructional activities from the same Chinese teacher. The experimental group received 14 updating training sessions over 4 weeks. The control group did not receive any specific training. Reading comprehension scales were conducted for both groups before and after training. Participants’ reading comprehension scales were reviewed and updating training was conducted in the double-blind experimental setting.

#### 3.1.3. Stimulus materials

(1) Updating training procedure

Brain imaging studies have confirmed that the N-back task better activates brain areas related to WM and executive functions (Richards et al., 2009). The Chinese character N-back training task and Chinese character stimulus materials from the Gao et al. (2023) experiment were used in this experiment.

(2) Chinese reading comprehension test

The Chinese reading comprehension test was selected from *The Big Rooster* (pre-test) and *A Favorite Picture* (post-test) of the Primary scholar’s Chinese Reading Ability Scales (Wen, 2005). Both were paper-pencil tests. The mean difficulty of the scales on the pre-test was 0.60 and on the post-test was 0.69. The discrimination of the pre-test was 0.30 and the post-test was 0.31. The scale contained three parts: micro reading comprehension, macro reading comprehension, and divergent reading comprehension. The scale internal consistency coefficient (Cronbach’s α) was *r* = 0.81.

#### 3.1.4. Procedures

(1) Chinese updating N-back training procedure

**FIGURE 1:**
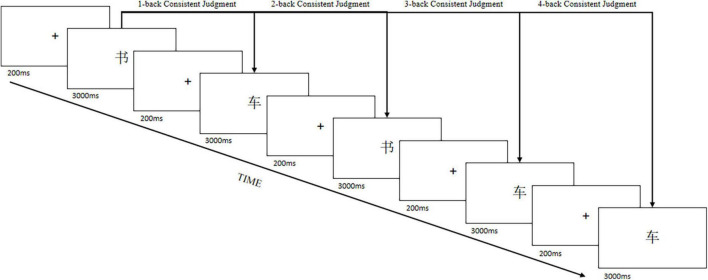
N-back training task.

The experimental group training procedure was presented on the computer. There were four difficulties of the training task: 1-back task, 2-back task, 3-back task, and 4-back task. Each difficulty contained 15 + n trials. As shown in Figure 1, the response time per trial was 3000 ms, and the participant was required to judge whether the current presented character was consistent with the previous nth presented character and responded with a key press. A total of 10 trials were inconsistent and five trials were consistent. All trials were randomly presented. Each participant was trained for 15 min each time, three to five times per week, for a total of 14 training sessions. The training was conducted in the school computer room. The participants were given a sticker as a reward at the end of each training session.

Each participant started with a 1-back task difficulty. In each difficulty, if the accuracy rate was higher than 80%, the participant entered the next difficulty; if the accuracy rate was lower than 80%, the participant stayed at that difficulty and retrained. There was only one chance to retrain, and if the retraining was not passed, the difficulty was dropped by one. The program ended automatically after 15 min of training for each participant.

(2) Reading comprehension scale

During the entire experimental period, the two groups of participants were administered both the pre-test and the post-test of the Primary scholar’s Chinese Reading Ability Scales. The pre-test was administered 1 week before the start of the training. The post-test was administered 1 week after the end of the training. The Primary scholar’s Chinese Reading Ability Scales comprised 100 points and lasted 30 min. Both groups underwent the scales simultaneously.

### 3.2. Results

#### 3.2.1. Updating training

**FIGURE 2:**
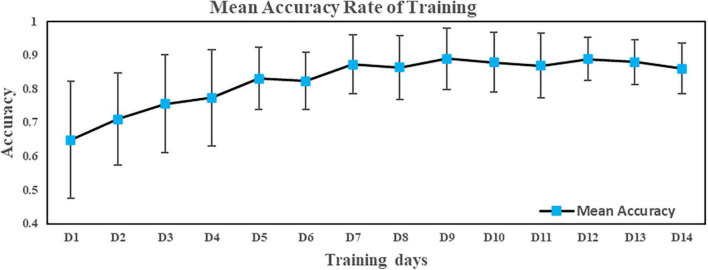
The mean accuracy rate of training in the experimental group.

The mean accuracy of the 14-day training for the experimental group is shown in Figure 2. To verify the effectiveness of the training, the mean accuracy of the first 3 days of training was compared to the last 3 days of training in a paired samples t-test. The mean accuracy rate on the last 3 days was significantly higher than that on the first 3 days [*t*_(34)_ = −11.07, *p* < 0.001, Cohen’s *d* = 2.18, *r* = 0.74].

#### 3.2.2. Chinese reading comprehension scales

The pre-test and post-test reading comprehension scores of the experimental and control groups are shown in Table 3. A 2 (participant type: experimental group vs. control group) × 2 (time: pre-test vs. post-test) repeated measures ANOVA of the total reading comprehension scale scores of the two groups revealed that the training failed to promote the total reading comprehension scores of the experimental group. The main effect of time on Chinese reading comprehension scale scores was significant [*F*_(1_,_71)_ = 6.78, *p* = 0.01, η_p_^2^ = 0.09]. The main effect of participant type was not significant [*F*_(1_,_71)_ = 0.34, *p* = 0.56, η_p_^2^ = 0.01]. The interaction effect of participant type and time was not significant [*F*_(1_,_71)_ = 0.20, *p* = 0.65, η_p_^2^ = 0.003].

**TABLE 3:** Pre-test and post-test reading comprehension scores of the experimental and control group.

Group	Pre-test					Post-test		
	**Micro reading**	**Macro reading**	**Divergent reading**	**Total score**	**Micro reading**	**Macro reading**	**Divergent reading**	**Total score**
Experimental group	13.25 ± 5.72	18.91 ± 8.22	16.99 ± 9.12	45.80 ± 16.84	12.71 ± 5.05	21.20 ± 11.72	15.69 ± 5.66	49.60 ± 17.50
Control group	13.16 ± 5.73	17.59 ± 6.63	16.79 ± 10.01	42.86 ± 16.61	8.95 ± 5.59	21.26 ± 9.97	18.05 ± 9.72	48.26 ± 18.60

A total of 2 (participant type: experimental group vs. control group) × 2 (time: pre-test vs. post-test) repeated measures ANOVAs were conducted for micro reading comprehension scores, macro reading comprehension scores, and divergent reading comprehension scores. The results of the analysis revealed changes in reading comprehension patterns after training in the experimental group. The repeated measures ANOVA for microscopic reading comprehension revealed a significant main effect of time [*F*_(1_,_71)_ = 7.50, *p* = 0.01, η_p_^2^ = 0.10]. A significant main effect of participant type [*F*_(1_,_71)_ = 4.02, *p* = 0.049, η_p_^2^ = 0.05]. A significant interaction effect of participant type and time [*F*_(1_,_71)_ = 4.47, *p* = 0.04, η_p_^2^ = 0.06]. Simple effects analysis showed no significant difference between the pre-test scores of the two groups of participants [*F*_(1_,_71)_ = 0.01, *p* = 0.94, η_p_^2^ = 0.00]. The experimental group scored higher than the control group in the post-test [*F*_(1_,_71)_ = 9.06, *p* = 0.004, η_p_^2^ = 0.11]. There was no change in the experimental group’s scores between the two tests [*F*_(1_,_71)_ = 0.19, *p* = 0.67, η_p_^2^ = 0.67]. The control group scored lower on the post-test than on the pre-test [*F*_(1_,_71)_ = 12.27, *p* = 0.001, η_p_^2^ = 0.15]. Because the post-test was more difficult than the pre-test, the decrease in the control group’s scores suggests a maintenance effect of training on the experimental group’s microscopic reading comprehension. The repeated measures ANOVA for macro reading comprehension scores revealed a significant time main effect [*F*_(1_,_71)_ = 5.31, *p* = 0.02, η_p_^2^ = 0.07]. The participant type main effect was not significant [*F*_(1_,_71)_ = 0.13, *p* = 0.72, η_p_^2^ = 0.002]. The interaction effect of participant type and time was not significant [*F*_(1_,_71)_ = 0.29, *p* = 0.59, η_p_^2^ = 0.004]. The repeated measures ANOVA for the discrete reading comprehension scores indicated that the main effect of time was not significant [*F*_(1_,_71)_ = 0.000, *p* = 0.96, η_p_^2^ = 0.000]. The main effect of participant type was not significant [*F*_(1_,_71)_ = 0.35, *p* = 0.56, η_p_^2^ = 0.005]. The interaction effect of participant type and time was not significant [*F*_(1_,_71)_ = 1.84, *p* = 0.18, η_p_^2^ = 0.03].

The above results indicate that updating training significantly improved the participants’ updating performance. However, the increase in updating performance could not change the total Chinese reading comprehension performance. Further analysis revealed that the increase in updating performance could change the participants’ reading comprehension patterns. This was reflected in the maintenance effect on microscopic reading comprehension when the difficulty of the reading task was increased (Hypothesis 2).

## 4. Discussion

Our study was divided into two experiments to explore the influence of CE on Chinese primary school students’ reading comprehension performance. Experiment 1 used the Chinese N-back task, the Chinese Stroop task, and the number-pinyin conversion task to verify the predictive effect of CE components on primary school students’ Chinese reading comprehension scores. Experiment 2 used Chinese character N-back training on Chinese primary school students based on the results of Experiment 1 to examine whether the enhancement of particular CE could promote the reading comprehension performance of the participants. We found that CE was a great predictor of Chinese reading comprehension for Chinese primary school students. Among them, updating had a prominent role in the prediction (Hypothesis 1). Further updating training of the participants revealed that the training did not improve the participants’ total scores on the reading comprehension scale but was able to maintain their microscopic reading comprehension scores on the more difficult post-test (Hypothesis 2).

The first result suggests that CE has a predictive effect on Chinese reading comprehension in Chinese primary school students. The findings of the present study align with previous research on the relationship between WM, especially CE, and reading comprehension (Huang et al., 2022; Linares and Pelegrina, 2023; Zaccoletti et al., 2023). Among these factors, updating plays a prominent role. This implies that CE, as a fundamental cognitive ability, plays a crucial role in how individuals process Chinese reading comprehension information. Our results are consistent with previous studies that have demonstrated a predictive effect of WM on English reading comprehension (Loosli et al., 2012). We provide ideographic evidence for a positive relationship between WM and reading comprehension from a Chinese reading comprehension perspective. The second result indicates that updating training enhances individual updating performance and demonstrates a maintenance effect on microscopic reading comprehension performance in more difficult tasks. This is consistent with findings from current research on the effects of WM training on reading comprehension (Artuso et al., 2019; Chang et al., 2019). We filled the gap of the lack of recruiting school-age children as participants and using ideographic characters as the content of the study, and the results suggest that updating training also affects the reading comprehension patterns of children. The experimental results also suggest that there may be an upper limit to the role of WM on reading comprehension.

Reading comprehension is one of the more complex and integrated processes in language acquisition. The reliance on WM is particularly prominent in reading comprehension. Individuals need to use the processing and storage functions of WM to process current information and to make connections between current information and general knowledge in long-term memory, and between current and prior information about the article, ultimately forming an overall mental representation of the article. At the same time, individuals also need to reveal the meaning of the reading material through activities such as expectation and reasoning. Therefore, during reading comprehension individuals need not only to have sufficient WM capacity, but also to maintain the continuous stability and flexibility of their WM operations (Muijselaar and de Jong, 2015). Those with high and low WM capacities have low WM capacity occupancy and low WM load when processing elementary, simple reading material, and there is no significant difference in reading comprehension between the two WM capacities. When processing advanced, complex reading material WM capacity was occupied heavily and WM load surged. The advantage of high WM capacity individuals was reflected in higher reading comprehension performance than low capacity individuals. At this time, high-capacity readers not only had sufficient WM capacity and higher processing storage space, but also could better focus their attention on reading. This facilitates high-capacity individuals to grasp the core information of the text and to process the reading material in detail. In contrast, low-capacity individuals exhibit poor attentional focus ability and tend to be attracted to irrelevant information. They can only use quick skimming and guessing to complete reading (Cowan, 1999; Peng et al., 2013).

However, at the primary school reading comprehension learning stage, reading comprehension content is at a relatively simple and elementary level. The difficulty of the reading material follows the same order of difficulty of learning: character–word–sentence–paragraph–chapter. Compared to the advanced and complex reading comprehension process, which requires complex processing of the information in the reading material and relies on the WM capacity, this elementary and simple reading comprehension process requires a continuous and stable regulation of the information in the WM. This ensures the coherence and integrity of the reading comprehension process. This means that there is a higher level of dependence on the continuous conditioning of WM content that is responsible for updating. This idea is supported by neuroimaging, where researchers found that the joint activity of the fusiform middle gyrus and precuneus gyrus in individuals after WM training suggests that WM reinforces Chinese language learning (Opitz et al., 2014). The continuous and stable modulation of updating provides a new perspective on the role of an individual’s general cognitive ability on reading comprehension: when individuals deal with complex language issues, higher levels of updating can make it easier for those with low WM capacity to use block strategies. That is, integrating words, phrases, and sentences into a block (WM unit) for processing and storage in WM. Thus, the limitation of WM capacity can be overcome. Then, updating training to enhance individual updating level during the critical period of CE can not only lay the foundation for the formation of block strategies in future higher stage reading comprehension activities, but also enhance the stability and flexibility of continuous regulation of WM. In turn, it can reduce the loss rate and error rate of information processing and storage in the current primary reading comprehension stages, and improve the individual’s microscopic reading ability. This would also release some of the WM capacity for alleviating the competition for resources in character-word-sentence comprehension, continuous processing of textual material, reasoning about the meaning of the text, and forming coherent and complete representations of the text in reading comprehension (Peng et al., 2013; Doyle et al., 2018).

## 5. Limitations and prospects

Participants from the same group (same school, instructional teacher, and community) were selected to balance the participants’ reading comprehension performance, language experience, and other factors. Firstly, the similarity of this group of participants in terms of instructional processes, reading strategies, and experiences may be responsible for their developing similar patterns of reading comprehension. These limitations may also be the main reason why the results of current research on the relationship between WM and reading comprehension remain controversial. Secondly, the balance of participant levels allowed us to analyze only the differences in participants’ reading comprehension scale scores, but failed to provide in-depth statistical test results. Thirdly, these considerations in the experimental design resulted in a small participant size for this experiment. It can only provide preliminary evidence for in-depth research in this field. Future studies with large samples across regions should be conducted so as to balance the effects of participants’ educational environments, reading strategies, learning motivation, and attitudes. Ultimately, this will provide persuasive evidence for the numerous debates among studies in this field.

In the updating training studies, it was found that the increased performance of WM positively transferred not only language acquisition processes such as reading and writing, but also individual fluid intelligence (Chein and Morrison, 2010; Zhao et al., 2013; Chen et al., 2018). This transfer effect suggests that WM, especially CE, may have a more fundamental role for the cognitive abilities and learning activities of individuals. It also suggests that individuals with a better level of WM before educational activities are an important prerequisite for successful learning activities. Then, in the future, further clarification of the mechanisms of WM in language learning with the help of brain imaging technology will provide an important basis for building a model of the role of WM in language learning and specifying the minimum level of WM required for successful language acquisition. It will also provide a new solution to the problem of balancing the learning time and learning efficiency of school-age children under the current education policy.

## 6. Conclusion

The present study assessed the predictive effect of the CE components of WM on the Chinese reading comprehension performance of Chinese primary school students by using the Chinese N-back task, the Chinese Stroop task, and the number-pinyin conversion task. The effect of updating training on Chinese reading comprehension of Chinese primary school students was further examined through Chinese character N-back training. We provide the first evidence of the predictive effect of CE on Chinese reading comprehension scores of Chinese primary school students. Of these, updating is the most important component. The second evidence we provide suggests that updating training, while not changing the total Chinese reading comprehension scores of Chinese primary school students in the scale, can have a maintaining effect on their Chinese reading microscopic reading comprehension scores. Combined with the currently limited literature on the effects of CE of WM on Chinese reading comprehension, these findings suggest that future research on the effects of CE of WM on reading comprehension should focus more on which specific aspects of reading comprehension are influenced by CE, rather than simply assessing the relationship between them. WM, especially CE, is an important cognitive ability that influences language learning, and such future work will provide us with a comprehensive understanding of the relationship between them.

## Data availability statement

The raw data supporting the conclusions of this article will be made available by the authors, without undue reservation.

## Ethics statement

The studies involving humans were approved by the Ethics Committee of the College of Psychology of Xinjiang Normal University. The studies were conducted in accordance with the local legislation and institutional requirements. Written informed consent for participation in this study was provided by the participants’ legal guardians/next of kin.

## Author contributions

JG: Conceptualization, Formal Analysis, Funding acquisition, Investigation, Methodology, Project administration, Resources, Software, Validation, Writing – original draft, Writing – review and editing. ZY: Data curation, Investigation, Project administration, Visualization, Writing – review and editing. FL: Data curation, Investigation, Project administration, Writing – review and editing. BY: Investigation, Project administration, Writing – review and editing. SW: Conceptualization, Supervision, Validation, Writing – review and editing.
